# A Common Approach to Low Vision: Examination and Rehabilitation of the Patient with Low Vision

**DOI:** 10.4274/tjo.galenos.2018.65928

**Published:** 2019-04-30

**Authors:** Esra Şahlı, Aysun İdil

**Affiliations:** 1 Ankara University Faculty of Medicine, Department of Ophthalmology, Low Vision Rehabilitation and Research Center, Ankara, Turkey

**Keywords:** Low vision, low vision rehabilitation, assessment of residual visual functions, assessment of residual functional vision, low vision aids

## Abstract

Due to the increasing age of the global population, rates of visual disability are increasing. Visual rehabilitation is an effective method for increasing quality of life among individuals with low vision or blindness due to unpreventable or untreatable causes. The goal of low vision rehabilitation is to produce people who are independent, have an economically viable profession or skill, and are able to enjoy their lives. The stages of modern low vision rehabilitation include the intake interview, assessment of residual visual functions, assessment of residual functional vision, interventions and recommendations, and vision rehabilitation therapies.

## Intraduction

According to World Health Organization (WHO) data from 2010, there were an estimated 285 million people living with visual impairment worldwide. Of these, 39 million were reported as blind and 246 million as having low vision. The most common causes (80%) of these visual impairments are treatable conditions such as uncorrected refractive errors and cataract. These are followed by age-related macular degeneration (AMD), glaucoma, and diabetic retinopathy. It has been reported that 65% of visually impaired and 82% of blind people are 50 years of age or older. Considering that the population is aging, this suggests that more people will be at risk in the future.^1^

Definitions of low vision and blindness may vary between countries. According to the definition accepted in the USA, best corrected visual acuity less than or equal to 20/200 in the better eye or a visual field less than or equal to 20° in the better eye is considered legal blindness.^2^ In the 2016 version of the International Classification of Disease (ICF)-10, visual impairment is classified in 5 categories based on presenting visual acuity. While older definitions were based on best corrected visual acuity of the better eye, the current definition is based on presenting visual acuity (with glasses if any, without glasses if not) in order to emphasize the burden of uncorrected refractive errors (Table 1). According to this, presenting visual acuity in the better eye equal to or better than 6/18 is defined as mild or no visual impairment; equal to or better than 6/60 and worse than 6/18 as moderate visual impairment (category 1); equal to or better than 3/60 and worse than 6/60 as severe visual impairment (category 2); and worse than 3/60 as blindness. Blindness is also separated into 3 categories: visual acuity worse than 3/60 (category 3), worse than 1/60 (or counting fingers at 1 meter) (category 4), and no light perception (category 5).^3^ According to this classification, those with moderate and severe visual impairment (visual acuity worse than 6/18 and equal to or better than 3/60) and those with a visual field less than or equal to 20° are defined as having “low vision” and require rehabilitation. Functionally, low vision can be regarded as a level of vision that prevents someone from performing their everyday activities. Having a presenting visual acuity worse than 3/60 and a corresponding visual field smaller than 10° is defined as blindness.^3^ Because this new definition also includes uncorrected refractive errors which were previously unaccounted for, the prevalence of blindness in various countries increases to 15% in all age groups and 25-30% among older adults. Studies have shown that the prevalence of low vision is up to 60% among older adults.^4,5,6,7,8^

The prevalence and causes of blindness and low vision in different societies vary based on their level of development. According to WHO data, the prevalence of blindness is 7.3/1000 in Africa, 3.5/1000 in the USA, 8.5/1000 in the Eastern Mediterranean Region, 3.0/1000 in Europe, 6.9/1000 in Southeast Asia except India, and 5.3/1000 in the Western Pacific Region except China. Global data indicate there are 3 people with low vision for each blind person; in the USA and Europe, which have the lowest rates of blindness, the prevalence of low vision is 25.6 and 28.7 per 1000, respectively. This rate is 25.4/1000 in Africa and 32/1000 in Southeast Asia.^9,10^

According to data from 2000, it was estimated that there were 937,000 (0.78%) blind and 2.4 million (1.98%) people with low vision over 40 years of age in the USA. Age-related macular degeneration (AMD) is the most common cause of blindness among Caucasians, accounting for 54.4% of cases. By 2020, the prevalence of blindness in the USA is predicted to increase by 70% to reach 1.6 million, and a similar increase is expected in the low vision population.^11^

**Figure 1:**
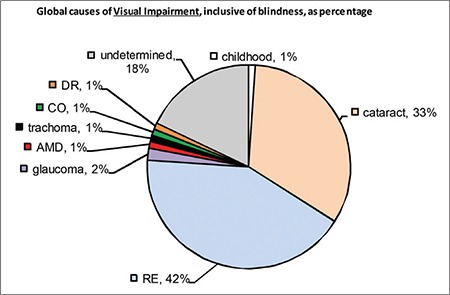
Distribution of global causes of visual impairment (taken from WHO report entitled Global Data on Visual Impairments 2010) RE: Refractive errors, AMD: Age-related macular degeneration, CO: Corneal opacity, DR: Diabetic retinopathy

**Figure 2:**
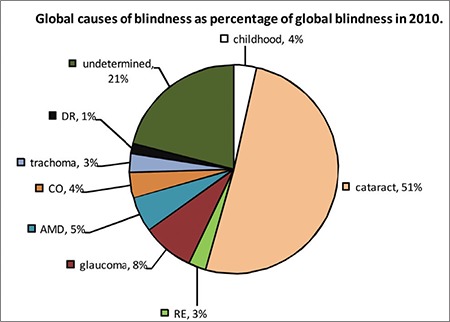
Distribution of global causes of blindness (taken from WHO report entitled Global Data on Visual Impairments 2010) RE: Refractive errors, AMD: Age-related macular degeneration, CO: Corneal opacity, DR: Diabetic retinopathy

Globally, 42% of visual impairment is due to uncorrected refractive errors, while 33% is caused by cataract. Other major causes include glaucoma, diabetic retinopathy (DR), trachoma, AMD, and corneal opacities. The primary cause of blindness is cataract (51%) (Figures 1 and 2).^1^ In North America and other developed countries, the main causes of vision loss are AMD, DR, and glaucoma. Other causes include herpes simplex keratitis, retinal detachment, retinal vascular diseases, and hereditary retinal degenerative diseases. In developing countries, the primary causes of vision loss are uncorrected refractive errors and cataract, followed by glaucoma, infectious diseases, injuries, and xerophthalmia.^12^ In short, visual impairment in developed countries is a result of unpreventable and/or currently untreatable causes, whereas preventable (infectious, e.g. trachoma, or nutritional, e.g. vitamin A deficiency) and/or treatable (e.g. cataracts) causes still play a major role in developing countries. The fact that most of the diseases that cause blindness and low vision are preventable or treatable has prompted many organizations to take action, especially WHO. According to the VISION 2020 report from WHO, low vision prevention and rehabilitation are among the primary global goals.^9^

A person’s ability to perform important sight-based tasks is defined as “visual functioning”. Reduced visual functioning due to disorders of the eye or visual system results in low vision. In addition to visual acuity, visual functioning should be assessed using parameters such as visual field, contrast sensitivity, electrophysiological tests, adequacy of preferred retinal locus, color vision, binocularity, and stereopsis.

Low vision rehabilitation aims to increase quality of life by enabling patients to live independently, have a vocation or skill with which they can financially support themselves, and enjoy life. The stages of modern low vision rehabilitation include the intake, assessment of residual visual function, assessment of residual functional vision, interventions and recommendations, and vision rehabilitation therapy.^13^

## 1. The Intake

The purpose of low vision assistance and rehabilitation is to enable individuals to perform the sight-based activities they want to do but currently cannot, using special methods and/or equipment.

The initial interview is of key importance, as it will influence the entire rehabilitation process. The patient’s family members should also be involved in some parts of this process, and it is imperative that sufficient time be allocated. History-taking from a patient with low vision differs from that in the classical ophthalmologic examination. The patient’s sociocultural characteristics, medical and ocular history, priorities, and goals must be questioned in detail and recorded. A patient is asked which tasks are difficult or impossible for them to perform in order to gain insight into their visual functioning. In particular, they should be asked about which activities they are limited in and wish to continue doing. It should be determined whether they use any methods to help them perform the activities that they have difficulty with. The environmental conditions in locations such as their home, school, and workplace should be questioned, as well as what provisions are needed to increase their visual functioning in these places.

It must be kept in mind that patients may have different needs, and each patient should be offered personalized solutions. Visual needs important to the patient may include reading, doing crafts, watching television, seeing the board in school, or reading road signs or bus numbers. Some patients can have unrealistic expectations of low vision rehabilitation, such as being able to drive. Although rehabilitation has a high success rate in regaining abilities such as reading, patients with low vision are not eligible to receive a driver’s license in Turkey. It may be necessary to inform patients what expectations are realistic without being discouraging. In cases where the patient and their family cannot adapt to their current situation and are pessimistic, the negatives of the patient’s visual impairment and disease should not be emphasized during the interview; instead, they should be guided and encouraged about what they can do.

When planning the rehabilitation program, questionnaires and scales about activities of daily living can be used to determine in detail what difficulties the patient faces in daily life. These scales are also used to evaluate the effectiveness of low vision rehabilitation. One of these scales is the Low Vision Quality of Life Questionnaire (LVQOL), developed by Wolffsohn and adapted to Turkish by Idil et al.^21^ and another is the National Eye Institute Visual Functioning Questionnaire (NEI-VFQ 25), which was adapted to Turkish by Toprak et al.^15^ The purpose of these scales is to characterize and determine the impact of visual impairment in daily life. The Turkish version of the LVQOL consists of a total of 24 items in 5 dimensions, including 12 items about distance vision, mobility, and lighting, 3 items about adjustment, 5 items about reading and fine skills, and 4 items on activities of daily living. The NEI-VFQ 25 comprises 25 items in 11 subgroups and 12 optional items. This scale includes items assessing general vision, difficulty in activities requiring near and distance vision, limitations of peripheral and color vision, ocular pain, vision-related limitation of social functions, role limitations, dependency, mental health symptoms, and driving difficulties, and general health. Higher scores in these scales correspond to better quality of life.^14,15^ When evaluating the patient with low vision, quality of life scales are useful for assessing the patient’s perceptions of their disease and whether rehabilitation has resolved their vision-related problems.

## 2. Assessment of Residual Visual Functions

Examination should be performed after identifying the patient’s priorities. Determining visual function is essential when examining the patient with low vision. Low vision examination differs from routine ophthalmologic examination in some respects. Distance and near visual acuity are assessed in detail. Best visual acuity should be determined with the most appropriate correction, because the patient’s residual vision will inform the selection of rehabilitation methods.

### Measurement of Distance Visual Acuity

Visual acuity measurement is the easiest and most useful method of assessing visual functioning, although it does not fully reflect a low vision patient’s performance in daily life. At this stage, it is essential to use charts that a person with low vision can see and place them at an appropriate test distance. A person with low vision gaining the ability to read some letters on a suitable and correctly applied chart when they could not read any letters in previous examinations is an important positive initial experience in the rehabilitation process. Accurate determination of visual acuity in a patient with low vision is also important to monitor disease, determine the amount of magnification needed for glasses or other optical device, evaluate response to therapy if provided, and to create disability reports if required. Examination should be performed under standard conditions (e.g., fixed chart distance and lighting) and with suitable charts. The Snellen chart is not appropriate for examination of the low vision patient because it has low sensitivity in the 6/10-6/24 range due to its irregular geometric arrangement and because the top lines are easier to read due to the crowding phenomenon. Instead, the logMAR-based Bailey-Lowie or Early treatment diabetic retinopathy study charts are preferred. Advantages of these charts are that they use logarithmic scales and the lines include equal numbers of letters of similar legibility. The spacing between the letters and lines is determined based on the size of the letters in each line. More lines are included at the low vision levels. Visual acuity is scored as 0.1 logMAR for each line and 0.02 logMAR for each correctly read letter. Better visual acuity corresponds to a lower logMAR score.^2,16^ Depending on visual acuity, measurement can be performed by adjusting the distance between the patient and chart to 2 meters or even 1 meter. It is suitable for use in low vision examination because it provides more sensitive measurement at low vision levels, facilitates refraction examination, and is preferred for academic purposes.

### Measurement of Near Visual Acuity

For patients with low vision, charts that include text samples are better for assessing near vision than charts that use optotypes. This enables the evaluation of reading performance, detection of any scotomas, and assessment of the effectiveness of therapy or rehabilitation. During examination, it must be ensured that the distance between the individual and the near vision chart is appropriate and fixed. The patient’s near vision is measured monocularly and binocularly using an addition suitable for the working distance of the reading chart. The metric M-unit is used for letter size. Near vision acuity is recorded as reading distance in meters divided by letter size in M-units. The Minnesota Low Vision Reading Chart (MNREAD), which can be applied using a computer screen or printed cards, is one of the charts frequently used in patients with normal or low vision, especially for international comparisons.^16^

### Refraction Test-Retinoscopy

Refraction testing must be performed more carefully in a patient with low vision. When examining low vision patients with abnormal head position, eccentric gaze, or nystagmus, the use of trial frames and lenses should be preferred over phoropter.

For patients with eccentric fixation or nystagmus and for uncooperative patients, cycloplegia and dynamic retinoscopy should be performed when measuring refractive error. Although refractive error can be measured using an autorefractometer in patients with low vision, determining refractive error by retinoscopy is ideal. When a clear reflection cannot be obtained, the patient should be approached until a reflection is seen, and necessary adjustments should be made based on this distance. After retinoscopy, the patient’s refractive error is confirmed using subjective methods such as fogging and cross-cylinder.

Remarkably, for approximately 15% of patients referred for low vision rehabilitation, functional vision can be restored by simply prescribing appropriate distance and/or near vision spectacles.

### Visual Field

Visual field is one of the most important parameters of visual function in the low vision patient. Diseases involving the macula, such as AMD, hereditary macular dystrophies, and macular edema, lead to scotomas that significantly impact visual functioning and reading performance. The Amsler Grid test is especially useful for identifying the location and size of central scotomas. However, this test is inadequate for small scotomas and conditions such as macular diseases in which fixation is commonly extrafoveal and unstable. These types of visual field defects are best evaluated by scanning laser ophthalmoscope (SLO). Because SLO provides instant retinal images, visual field defects and the related area of the retina can be evaluated simultaneously. Microperimetry using SLO technology enables the detection of important parameters such as preferred retinal locus and fixation stability in low vision patients with central scotoma, and trained retinal locus training can be provided.^2,16^

Peripheral visual field loss adversely affects an individual’s orientation in unfamiliar environments, mobility, and hazard perception. These defects are often seen in patients with advanced glaucoma and retinitis pigmentosa. Kinetic (Goldman) and static (Humprey, Octopus) perimetries can be used to evaluate such defects.^2,16^

High-power prism designs are used for hemianopias and quadrantanopias of neurological origin and cases of tunnel vision due to diseases such as retinitis pigmentosa.^17^

### Assessment of Contrast Sensitivity

Contrast sensitivity is the power to distinguish differences in shade between two regions. Although contrast sensitivity tests are not used in clinical practice for every patient with low vision, they can be performed for patients whose visual functioning is poorer than expected based on their measured visual acuity. Clinically, deficits in contrast sensitivity are especially common in corneal edema, cataract, optic nerve diseases, and some retinal diseases. Patients with low contrast sensitivity might require more magnification than expected for their visual acuity and may benefit from increased ambient light. Closed-circuit television systems that increase contrast and broaden the visual field can be recommended to these patients.^2^ Many contrast sensitivity charts are used in clinical practice to measure perceived contrast, such as the Vistech VCTS test, Pelli-Robson Letter chart, Arden chart, CSV-1000 chart, and Regan chart. For patients with low vision, contrast sensitivity tests designed specifically for low vision should be used, such as the CSV-1000LV, ELCT, and CSV-1000 1.5 cycles/degree.

### Color Vision

Hereditary and acquired color vision disorders have several distinguishing features. Hereditary color blindness (protanopia and deuteranopia) is a stable, binocular, usually red-green color vision deficiency that preferentially affects males. Other visual functions are normal. Acquired color vision deficiencies can be monocular and asymmetrical, are often progressive, and usually involve blue-yellow color blindness. Most color vision disorders in patients with low vision are blue-yellow dischromatopsia. Pseudoisochromatic plates are the most commonly used color vision tests. They are simple and can be performed quickly. They comprise colored numbers or paths on a background of equal saturation. The Ishihara test, the most well known pseudoisochromatic table, only tests red-green vision. For blue-yellow dischromatopsia, which is more common among patients with low vision, color arrangement tests such as the Farnsworth 100 Hue and D 15 tests or the Wang & Wang color vision plates are more appropriate than the Ishihara test. In addition, the reliability of pseudoisochromatic tests decreases at visual acuity levels lower than 6/20. In general, blue-yellow color blindness is considered to be associated with large lesions involving the outer retina, while red-green color blindness occurs in lesions involving the inner retina and optic nerve. Furthermore, blue-yellow color blindness is seen in cataract and glaucoma, while red-green color blindness occurs in cone dystrophy. As part of rehabilitation, patients can be advised to seek high color and tone contrast.^16,18^

### Glare Test

Glare refers to excessive brightness in the visual field and can be accompanied by asthenopia, headache, and squinting. Glare can be associated with media opacities such as cataracts and corneal scar, or albinism, achromatopsia, or aniridia. It can be assessed simply during visual acuity measurement by holding a light source near the fixation line and observing the reduction in the number of lines or letters the patient can read.^2,16^

## 3. Assessment of Residual Functional Vision

Low vision patients with similar residual visual functions may have very different performance when it comes to utilizing their vision. Assessment of residual functional vision determines how and to what extent the low vision patient can use their residual vision and the individual and environmental factors that affect this ability. This also includes educational vision assessment to facilitate appropriate education planning.

As explained in detail in ICF system, in addition to their visual functions, an individual’s activity and participation and environmental factors must be evaluated in a rehabilitation program. In other words, visual functions determine a person’s capacity, whereas functional vision refers to their performance.^19^

Therefore, functional vision assessment identifies how the patient uses their vision and what visual skills and environmental adjustments they need to better use their vision. It is based on the patient’s actual performance in the target activity and measurement of the adequacy of this performance. For example, in a patient whose primary goal is to read, residual visual function is determined using methods such as visual acuity, refractive error, and visual field, while residual functional vision is measured using a performance index such as reading speed. Reading performance should be assessed using continuous text cards instead of solitary optotypes. Continuous text cards must be representative of commonly read materials and commonly used words in the population, be standardized in terms of length and width, and be printed in the native language of the population.

**Figure 3:**
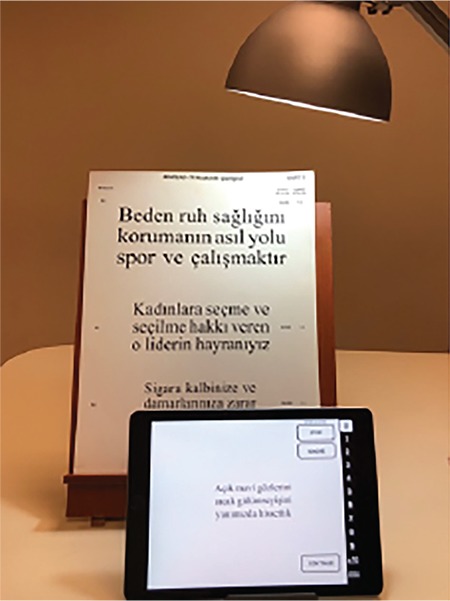
Assessment of reading performance using MNREAD cards

An objective measure of reading performance is maximum reading speed. Other parameters that can be used in assessment include reading acuity, critical print size, and Reading Accessibility Index. Maximum reading speed is the reading rate that is not limited by print size. Reading acuity is the smallest print size that can be read without making any errors; critical print size is the smallest print size that can be read at maximum speed. The recently developed Reading Accessibility Index indicates the visual accessibility of familiar printed material and is calculated as the mean reading speed across the ten largest print sizes on an MNREAD chart. It represents reading performance in daily life.^20^ MNREAD cards, developed at the University of Minnesota, can be used to assess reading performance. They provide corresponding values for reading acuity in Snellen, logMAR, and M-units from 40 cm. Although originally in English, they have been validated in various languages. A Turkish version has also been developed and validated and is of equal difficulty to versions in other languages to allow its use in international studies (Figure 3).^21^

Quality of life scales can be used in the subjective evaluation of functional vision. It is also possible to evaluate the effectiveness of rehabilitation with these scales.

Daily visual goals usually include reading, writing, watching television, dressing, performing personal care, moving around, cooking, doing home maintenance, cleaning, and working. A rehabilitation program is designed taking into account the patient’s visual priorities and their distance, near, or intermediate distance vision needs. This planning requires a multidisciplinary low vision team. In order to increase the patient’s participation and motivation, their family should be involved in planning and implementing the low vision rehabilitation.

## 4. Interventions

The data obtained in the first three stages are evaluated and an individualized intervention program is planned for each low vision patient. This program encompasses the necessary techniques and/or assistive technology.

### Devices Used in Low Vision Rehabilitation

### Optical systems

### I. Telescopes

**Figure 4:**
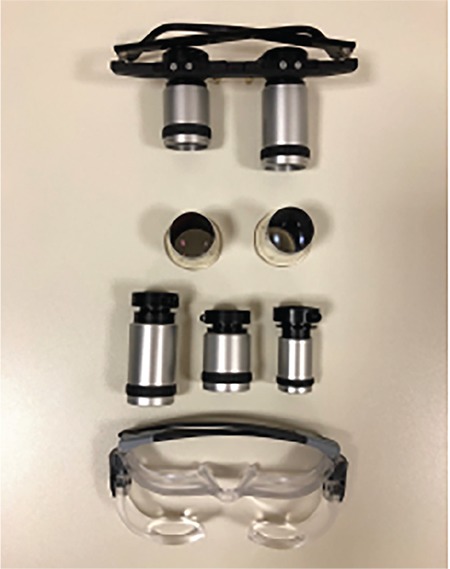
Some types of telescopes used in our clinic

Advantages of telescopes include being able to magnify an image at long working distances with hands-free use; however, they also have disadvantages such as being difficult and dangerous to use when moving due to narrowing of the visual field, causing difficulty in achieving binocularity, and being costly, and they can also cause esthetic concerns. They can be integrated into the patient’s own prescription glasses, and some models are also focusable (Figure 4). Their length increases with their magnifying power, and visual field narrows as their length increases and diameter decreases. Bioptic telescopes can be used at magnifications of up to 6x. When a telescope is prescribed for a patient with low vision, they must be trained in its use.

Telescopes are either Galilean or Keplerian depending on their optical principles. The Galilean telescope consists of two lenses, a low-power plus objective lens and a high-power minus eyepiece lens, and it gives an upright image. The Keplerian telescope also consists of two lenses, a low-power plus objective lens and high-power plus eyepiece lens. The inverted image obtained with Keplerian telescopes is corrected with prisms. Although Galilean telescopes have certain advantages such as being shorter and lighter and having a larger visual field, Keplerian telescopes have better image quality because they use light more efficiently. Keplerian telescopes are more complex with a wider range of focus. The telescopes used in low vision rehabilitation are usually Keplerian.^22^

Telescopes can be focusable or fixed-focus depending on their focusing characteristics. In focusable telescopes, the patient’s spherical error can be corrected and a base lens may be required for high astigmatism. With fixed-focus telescopes, the patient’s refractive error (spherical + cylindrical) must be given as the base lens.

Depending on the patient’s vision level, telescopes can be prescribed monocularly or binocularly. For near vision, a +3.00 to +12.00 D cap (reading cap) can be attached. Telescopes can be hand-held, clip-on, or spectacle-mounted (large-scale, bioptic, mini-telescope). Spectacle-mounted telescopes are mostly used for watching television or by school-aged children for looking at the board, whereas a monocular hand telescope is hung around the neck and used only when needed, allowing the user to continue their everyday activities.

**Figure 5:**
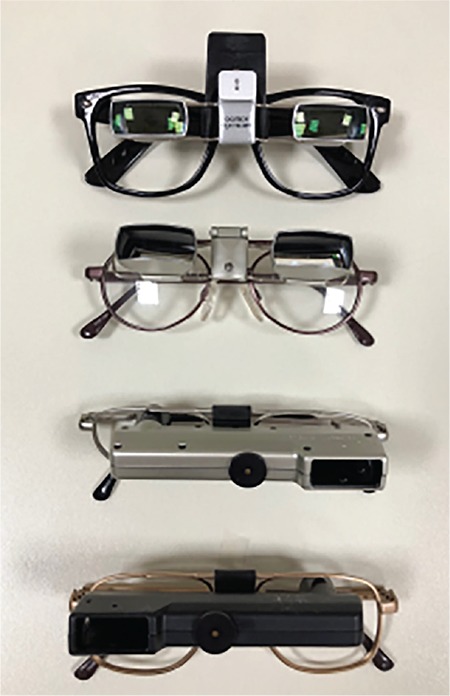
Various examples of bioptic telescopes

Although telescopes are not suitable for use when moving due to narrowing of the visual field, various special designs have been developed in an effort to overcome this limitation. These designs, which are used when in motion, are bioptic telescopes and autofocus telescopes. In bioptic telescopes, a compact, low-power magnifying telescope is placed in an area in the patient’s visual field, usually the superotemporal region. When the patient looks through their glasses, the magnified image from the telescope can be viewed when needed by adjusting their head or eye position. With autofocus telescopes, this process is modified with a motorized focusing system so that the user can easily follow objects at different distances (Figure 5).^22^ Although bioptic telescopes are useful for distance viewing, their use is limited by their appearance and the ring scotoma surrounding the magnified image. This led to the recent development of ‘in-the-spectacle-lens’ telescopes, a design in which a wide-field Keplerian telescope is built completely within the spectacle lens. By simultaneously using the magnified and nonmagnified view of the viewing area, the vision multiplexing feature provided by these devices facilitates the patient’s orientation and navigation.^23^

### II. Microscopes (High-Diopter Near Spectacles)

**Figure 6:**
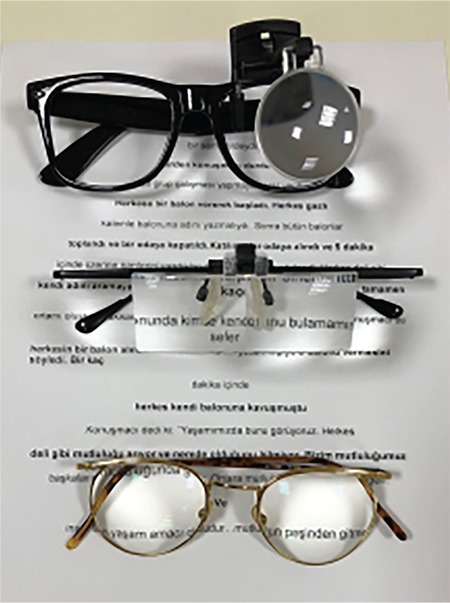
Some types of microscopes used in our clinic

After correcting hyperopia, near addition and reading distance are calculated according to Kestenbaum’s rule. For example, in a patient with a corrected visual acuity of 20/100, the near add is the inverse of visual acuity, 100/20=5 D, and near reading distance is 1/5=20 cm. The add is gradually increased to the dioptric power that allows the patient to comfortably read a text size of 1 M. The actual value will be higher than the predicted value in patients with low contrast sensitivity or macular scotoma and those who want to read letters smaller than 1 M. Binocular vision up to +10 D is possible. As the dioptric power increases, reading distance is reduced accordingly. If the reading distance is too short, it can be increased with high-power plus lenses held away from the eye with special clip-on systems. The effect of additional illumination must also be assessed during examination. The advantages of microscopes include their wide visual field, hands-free operation, and pleasing esthetic appearance. Negative aspects are their short working distance and inability to tolerate values greater than 10 D binocularly (Figure 6).^2,24^

**Figure 7:**
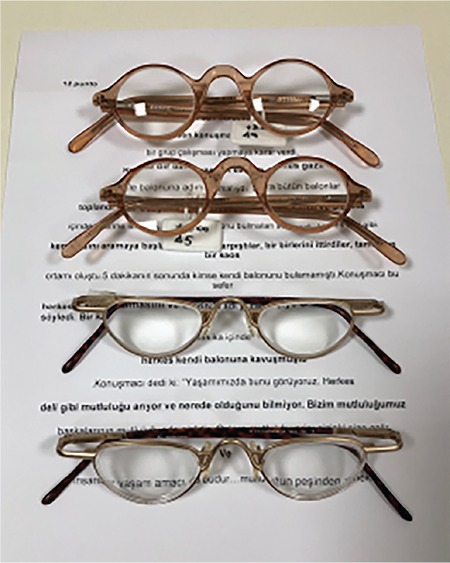
Microscopes with prism additions

In patients whose binocular vision is better than their monocular vision (i.e., with similar visual acuity in both eyes), a base-in prism can be added to facilitate accommodative convergence (Figure 7). Although there are various formulas to calculate Δ addition, a base-in Δ roughly twice the D power addition can be added to both eyes. If the patient’s reading performance is better when their less sighted eye is closed (i.e., the patient is functionally monocular), a frosted lens can be prescribed for the less sighted eye or the patient can be instructed to close the weaker eye when reading.^24^

Because high D (greater than +4.5 D) additions in bifocal and progressive lenses are difficult to tolerate binocularly and the likelihood of problems at intermediate and far distances increases in parallel with D power, dedicated reading glasses should be recommended to patients with low vision. Moreover, as the use of near vision spectacles provides a larger visual field, it will enable eccentric viewing.

### III. Magnifiers

Magnifiers can be used in addition to near vision spectacles in order to meet the needs of low vision patients when reading and performing tasks requiring near vision. They can be used simultaneously with near vision spectacles, and do not require myopic correction in most patients. Remember that with magnifiers, the greater the working distance, the smaller the visual field. Magnifiers are available as hand-held, stand, illuminated, fiberoptic, and dome/bar magnifiers.

**Figure 8:**
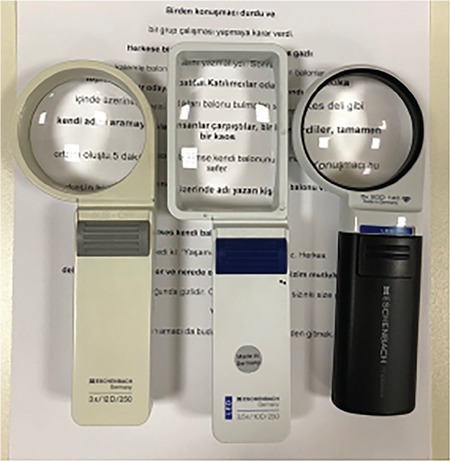
Hand-held magnifiers

Advantages of hand-held magnifiers are that they are portable, can be used at longer working distances than spectacles, and are inexpensive. Some have built-in illumination. The virtual image can be brought closer to the focal plane at the back of the eye by changing the object distance. Aspheric magnifiers provide better image quality. They are useful when looking at mobile phone screens and price tags while shopping. However, they must be held steady at a fixed working distance (Figure 8).^25^

**Figure 9:**
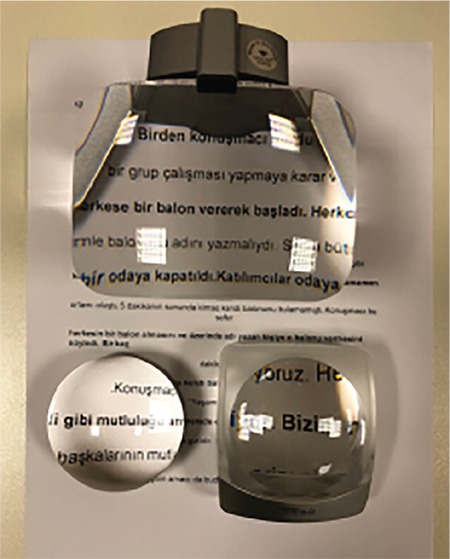
Stand magnifiers

With stand magnifiers, the object distance can be adjusted easily. They require a fixed, flat surface and usually include a built-in light source. This increases contrast and reduces the amount of magnification needed, thus increasing reading speed. Stand magnifiers should be used in conjunction with near vision spectacles of about +3.00 to +3.50 D in older patients. They may be preferable for those who cannot use hand-held magnifiers due to tremor, paralysis, arthritis, or poor hand-eye coordination, or those who require more magnification than spectacles provide (Figure 9).^26^

### IV. Filtering Lenses

**Figure 10:**
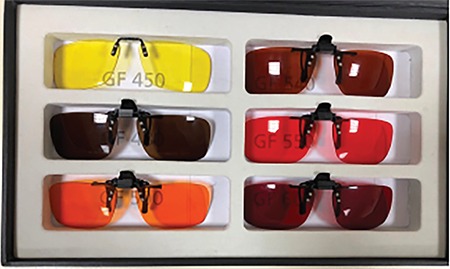
Filtering lenses

These lenses filter certain wavelengths of light while allowing the passage of other wavelengths. This reduces the patient’s photophobia and provides clearer vision by increasing contrast sensitivity. According to the patient’s needs, different filtering lenses can be prescribed for both indoors and outdoors. The lenses are different colors based on the wavelength they filter. Although there are filters recommended for certain diseases, it is more appropriate to try a set of filtering lenses to identify the filter the patient is most comfortable with (Figure 10).^27^

### V. Electro-optical Systems

**Figure 11:**
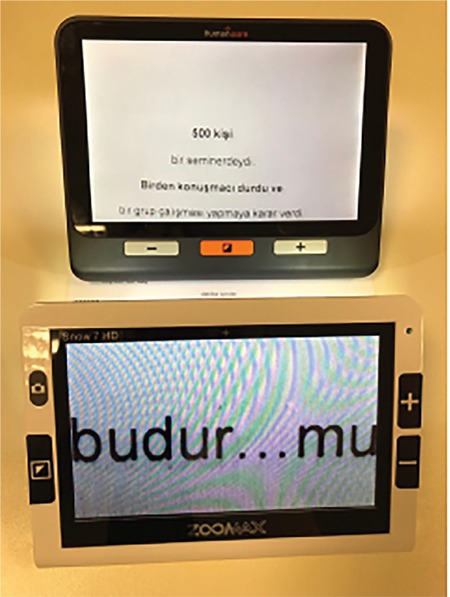
Examples of electro-optical systems

Closed-circuit television (CCTV) systems are systems that project visuals such as written text or images to a screen and enable adjustments such as magnifying the image and changing brightness and contrast. They are so called because of the direct cable connection between the camera imaging system and the display. Features such as variable magnification, auto-focus, magnification without focusing, reverse contrast, voice-command controls, and automatic forwarding have also been added to these systems. Electro-optical systems mitigate or overcome many problems associated with magnifying systems, such as narrow visual field, short working distance, reduced contrast, aberrations, and illumination. The main problem with electro-optical systems is that they are large and costly. However, with technological advances, systems now come in portable sizes and have become relatively less expensive (Figure 11).^2,28^

**Figure 12:**
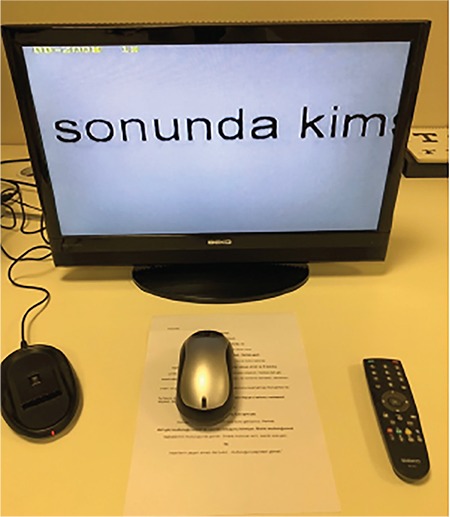
Mouse electronic magnifier

Mouse magnifiers are devices that look like a computer mouse and contain a camera that is moved over the material to be viewed. They are easy to carry, cheaper than CCTV systems, and can be connected to most personal computers. They can have variable magnification, reverse contrast, and focusing features. Their main disadvantage is limited viewing area (Figure 12).

Today, electronic tablets have become more popular than most optical systems due to their many functions and applications that assist those with low vision, especially school-age children. Most individuals with low vision can benefit from electronic reading devices such as the iPad (Apple, Cupertino, CA, USA) and Kindle (Amazon, Seattle, WA, USA).^29^ A prospective study showed that these types of electronic devices increased reading performance in most patients.^30^ These devices include applications that enable the user to increase the size and darkness of characters, adjust the contrast, brightness, and color of the display background, magnify and zoom, zoom by taking a photograph of an image, take spoken commands, and read text aloud. Their advantages include ease of access, relatively low cost, and combination of different functions that can be used for both distant and near tasks.^31^

### Non-Optical Systems

Non-optical systems increase the patient’s residual visual function or use signals that stimulate one of the other senses. Illumination, large-print books, increased contrast, typoscope, reading stands, and sunglasses or spectacles with filtering lenses to reduce glare can be used alone or in conjunction with optical systems in patients with low vision.

Illumination reduces the need for magnification and increases reading performance, particularly in macular degeneration patients who have reduced contrast sensitivity. Patients must be taught how to properly direct table lamps while reading. The built-in illumination in hand-held and stand magnifiers increases reading performance for most patients. On the other hand, patients complaining of excessive glare will benefit from reducing the light level and using hats, sunglasses, filtering lenses, and light-blocking glasses. Patients can be advised to increase the contrast when printing documents, try different contrasts such as a light-colored object on a dark background, use glasses with contrast-enhancing yellow or orange filtering lenses, and use a typoscope or electro-optical system.^2^

## 5. Recommendations and Vision Rehabilitation Therapy

As visual impairment progresses, patients can be offered alternate tools and techniques such as white cane training, use of the Braille alphabet, audio books, and voice recording devices. It is also very important to modify the patient’s living conditions. Taking measures such as sitting students in the middle of the front row of the classroom, organizing the kitchen and other home environments in a contrasting and appropriate way, and accentuating steps and handrails will make daily life easier. Vision loss can have a major impact on some quality of life and emotional state in some individuals. These people should receive psychological counseling to help them adjust and overcome the emotional problems they are experiencing.

Low vision rehabilitation is not just the prescription of a low vision aid. Training programs consisting of habituation exercises practiced in the clinic or at home constitute one of the most important stages of rehabilitation. Various training programs and courses are implemented in vision rehabilitation therapy to develop related functions and improve performance. Some of these programs are reading and writing skills, orientation and mobility, and driving education in countries where it is legal. Occupational therapists conduct assessments at the patient’s home, school, or workplace to improve orientation and mobility and facilitate adaptation. If there are target activities in the patient’s real life environments, they are also practiced using the auxiliary devices and the necessary environmental adjustments are recommended.^13^

## Figures and Tables

**Table 1:**
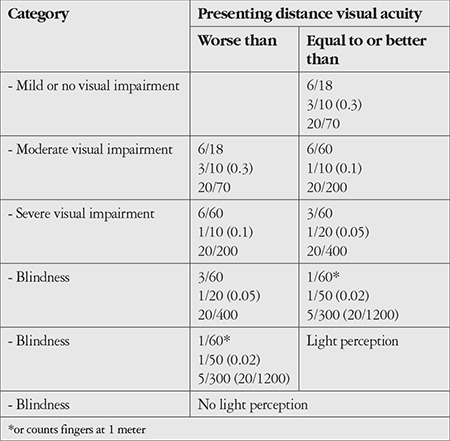
Classification of visual impairments according to the International Classification of Disease-10 2016 revision
